# Vascular access surgery training in the United Kingdom is currently perceived (by trainees) to be inadequate

**DOI:** 10.1308/rcsann.2023.0110

**Published:** 2024-04-02

**Authors:** T Richards, I Ahmed, R Harvey, K El Sakka

**Affiliations:** University Hospitals Sussex NHS Foundation Trust, UK

**Keywords:** Vascular surgery, Vascular access surgery, Arteriovenous fistula, Curriculum, Training

## Abstract

**Introduction:**

Vascular access surgery (VAS) involves the creation and maintenance of arteriovenous access to facilitate haemodialysis. The prevalence of haemodialysis is rising despite increases in kidney transplants on a yearly basis. There is currently only one access surgery fellowship accredited by the Royal College of Surgeons of England. We aimed to establish the experience and perceived competence in access surgery of senior vascular surgery trainees.

**Methods:**

A short questionnaire (SurveyMonkey) was used to survey all senior (ST6–ST8) vascular surgery trainees in Health Education England (HEE) vascular surgery training programmes. The short survey asked trainees to report their: (1) training grade; (2) training deanery; (3) experience of access surgery; and (4) whether senior trainees thought they would be able to independently undertake primary access surgery post-completion of training (post Certificate of Completion of Training). The survey was circulated via HEE deaneries and the vascular surgery trainees' society: the Rouleaux Club.

**Results:**

Twenty-eight senior (ST6–ST8) vascular surgery trainees responded to the survey: 29.6% were ST6 level, 33.3% were ST7 and 37.1% were ST8. Deanery respondence was evenly spread, although London was overrepresented (37.1%). In total, 28.6% had been involved in fewer than 10 cases, 35.7% in 10–25 cases, and 35.7% in more than 25 cases. Almost 54% of senior vascular surgery trainees believed they would not be able to undertake independent access surgery once they had completed training.

**Conclusions:**

Competence in access surgery is an increasing requirement of a consultant vascular surgeon. More formalised training is required to adequately train the next generation of vascular surgeons.

## Introduction

Vascular access surgery (VAS) involves the creation and maintenance of arteriovenous access to facilitate haemodialysis. Primary VAS is done either in anticipation of, or shortly after the requirement for commencement of haemodialysis, and generally includes radiocephalic and brachiocephalic fistulae in preference to the more complex brachiobasilic anastomoses and arteriovenous grafts.^1^ The prevalence of haemodialysis is rising despite increases in kidney transplants on a yearly basis.^2^

VAS was historically undertaken by transplant surgeons on an outreach basis, but has more recently shifted to local services generally provided by vascular surgeons at regional dialysis hubs. This evolution in service provision has resulted in variation in how the services are delivered, and how training in VAS is provided. Indeed, there is currently only one VAS fellowship accredited by the Vascular Society for Great Britain and Ireland,^3^ and national interest means that the fellowship is oversubscribed without capacity to formally train all those who express an interest.

The vascular surgery curriculum currently includes a requirement for ten procedure-based assessments, with four at level 4 as an outcome for the Certificate of Completion of Training (CCT).^4^ However, anecdotal reporting from trainees frequently includes a lack of experience in VAS resulting in the inability to undertake independent VAS as a newly qualified consultant.

This project aimed to formalise this reporting and ascertain the gap in the current requirement for experience required by the vascular surgery curriculum, and the reality encountered by trainees.

## Methods

A short questionnaire was created in SurveyMonkey and circulated to vascular surgery trainees. Respondents were limited to senior (ST6–ST8) trainees holding a national training number (NTN) in the United Kingdom (UK). Seniority was thought to be important, because more junior trainees may not yet have had exposure to VAS, but could reasonably anticipate competence at the end of their training. Staff-grade and other non-deanery registrars were excluded because of a heterogeneity of experience, and potentially absent outcome of perceived competence at certification.

The survey asked trainees to report their:

• training grade;
• training deanery;
• experience of access surgery (number of cases completed as performed [P], supervised trainer unscrubbed [STU], supervised trainer scrubbed [STS] or assisted [A]);
• whether senior trainees thought they would be able to independently undertake primary VAS post-CCT.

The survey was circulated via deaneries and training programme directors where possible and the vascular surgery trainees’ society (the Rouleaux Club). Further, ad hoc, circulation was pursued to attempt to maximise the number of respondents. Replies were collected between April 2022 and March 2023.

## Results

Twenty-eight senior (ST6–ST8) vascular surgery trainees responded to the survey: 29.6% were at ST6 level, 33.3% were ST7 and 37.1% were ST8 (Table 1). Deanery respondence was evenly spread, although London was overrepresented (37.1%) (Table 2). Some 28.6% of respondents had been involved in fewer than 10 cases, 35.7% in 10–25 cases, and 35.7% in more than 25 cases (Table 3).

**Table 1:** What grade are you?

	*n* = 27	%
ST6	8	29.6
ST7	9	33.3
ST8	10	37.1

ST = specialty trainee

**Table 2:** What is your training deanery?

	*n* = 27	%
East Midlands	2	7.4
East of England	3	11.1
Kent, Surrey and Sussex	3	11.1
London	10	37.1
North East	0	0
North West	2	7.4
South West	2	7.4
Thames Valley	0	0
Wessex	1	3.7
West Midlands	0	0
Northern Ireland	0	0
Scotland	1	3.7
Wales	1	3.7
Yorkshire and Humber	2	7.4

**Table 3:** How many vascular access cases have you been involved in to date (assisted/supervised trainer scrubbed/supervised trainer unscrubbed/performed)?

	*n* = 28	%
<10	8	28.6
10–25	10	35.7
>25	10	35.7

Some 53.6% of senior vascular surgery trainees believed they would not be able to undertake independent VAS once they had completed training (Table 4).

**Table 4:** Do you think you will be able to independently undertake primary vascular access surgery after your Certificate of Completion of Training?

	*n* = 28	%
Yes	13	46.4
No	15	53.6

## Discussion

### The problem

End-stage renal disease is increasing, and the waiting list for a kidney transplant is lengthening, with an increase of 43% in 2022 from the previous year and 4,643 adults on the UK active kidney transplant list (despite a 32% increase in the number of adult kidney-only transplants completed) creating an increasing demand for VAS.^5^ Although there are a number of minimally invasive endovascular techniques to create reliable access for haemodialysis that may not necessarily be performed by a vascular surgeon, these are currently prohibitively expensive, and require a significant cultural change in the management of patients with end-stage renal failure approaching dialysis.^6^ In the short term, at least, the demand for VAS is therefore likely to increase.

There are no data available for annual NTN numbers prior to 2022 when 26 NTNs were appointed. If the number of vascular surgery NTNs in 2022 is extrapolated to previous years, there would be approximately 125 current trainees (not including estimates of numbers undertaking research or other breaks in training, and likely an overestimation because NTNs have been increasing since application to vascular surgery training was separated from general surgery). Half of these would be ST6–ST8 and therefore included in the demographic for this study. This would provide an estimated response rate of 45% and a significant sample of a small cohort of trainees.

Even if all 28.6% of respondents who have been involved in fewer than 10 VAS cases were at ST6 level, this is still reflective of a lack of available training for a field of vascular surgery for which competence is required for CCT. It is unclear whether this gap in experience is due to a variety of local factors or a problem at a national level, but significant regional variation in the organisation of VAS is a likely notable contributor.

In total, 53.6% of trainees do not think they will be able to independently undertake primary VAS as a newly qualified consultant. If the survey was extended to include more complex surgery than primary forearm fistulae, the proportion would be expected to be even lower. In the context of increasing demand and ongoing culture shift from transplant to vascular surgeons undertaking VAS, this gap in requirement and competence can be expected to increase. It is unreasonable to assume that newly qualified consultants should expect to acquire expertise in VAS after appointment, and the shortfall is therefore likely to increase.

### The solution

Addressing the current perceived deficiency in training should focus on two areas.

First, and most problematically, the heterogeneity of provision of VAS should be standardised with an aligning of both how renal physicians refer to, and interact with, vascular surgeons in the management of patients requiring haemodialysis, and how these patients are managed by their surgical department. This would provide an opportunity to standardise training and ensure the majority of current vascular surgery trainees are able to provide a service that is expected to increase. This would not be a small undertaking and requires leadership at a national level.

Second, the vascular surgery curriculum requirement for competence should be perceived by training programme directors, departments and trainers as similar in importance to more established aspects of vascular surgery like aortic aneurysmal and symptomatic carotid disease, and lower limb revascularisation. In-house training should include formal VAS exposure, and where this is not possible, there should be regionally supported access to structured training for departments or units that regularly undertake VAS.

The success of the Brighton Vascular Access Fellowship demonstrates that appropriate resourcing and allocated time for trainees to immerse themselves in a novel field of vascular surgery can provide competence in a relatively short period (eight all-day lists plus exposure to clinic and multidisciplinary team meetings where possible).^3^ This model could be extended to other busy VAS departments to increase the national output of newly qualified consultants competent in, at least, primary VAS.

Transplant surgery has a long-established holistic method of training surgeons in VAS, with a focus on the holistic management of patients approaching the need for dialysis. In addition to the adoption of a similar methodology in vascular centres, there might be the possibility of vascular trainees completing fellowships in transplant units to contribute to the currently perceived lack of training opportunities.

## Conclusion

In the context of an increasing demand for VAS, there is a perceived shortfall in competence by current senior vascular surgery trainees. Improving the organisation of VAS provision and formalising training will contribute to the corporate competence of VAS in newly qualified vascular surgeons, addressing a deficit that is currently expected to increase.
